# Complex Pharmacodynamics of Aripiprazole and Haloperidol in Schizophrenia Management: A Case Report

**DOI:** 10.7759/cureus.71144

**Published:** 2024-10-09

**Authors:** Rihab Farooq, Huda Zahid, Ammara Shakil Sahibole, Syed Ali Bokhari, Mazin A Mukhtar

**Affiliations:** 1 Psychiatry, Al Amal Psychiatric Hospital, Emirates Health Services, Dubai, ARE; 2 General Medicine, Dubai Medical College for Girls, Dubai, ARE; 3 General Practice, GSM Medical Center, Dubai, ARE

**Keywords:** aripiprazole, cyp450 metabolism, dopamine d2 receptor, drug interaction, extrapyramidal symptoms (eps), haloperidol, partial agonist, schizophrenia

## Abstract

Schizophrenia is a complex psychotic disorder characterized by positive symptoms, such as hallucinations and delusions, and negative symptoms, including emotional withdrawal and apathy. Its profound impact on an individual’s functioning requires meticulous and sustained management, typically through the use of antipsychotic medications. While these treatments aim to alleviate symptoms and improve quality of life, long-term therapy often necessitates careful balancing to mitigate potential side effects. As a result, ongoing monitoring and personalized care are essential for effective management. This case report discusses a 44-year-old male with chronic schizophrenia who experienced worsening psychotic symptoms following an interaction between aripiprazole, a partial dopamine agonist, and haloperidol, a first-generation antipsychotic (FGA). Due to aripiprazole’s unique mechanism of action, his medication regimen was adjusted, discontinuing aripiprazole and transitioning him to risperidone 4 mg twice daily, valproic acid 500 mg twice daily (BID), haloperidol 10 mg twice daily, and quetiapine 200 mg once daily (OD). Following these adjustments, the patient showed significant improvement over the subsequent weeks and was discharged from inpatient care. This case underscores the unexpected consequences of combining two medications and highlights the challenges posed by aripiprazole’s mechanism of action. It emphasizes the importance of vigilant monitoring and careful drug selection when managing schizophrenia, particularly in cases involving complex pharmacological profiles.

## Introduction

Schizophrenia is a severe psychiatric disorder that affects approximately 1% of the global population, characterized by both positive symptoms, such as hallucinations and delusions, and negative symptoms, including social withdrawal, affective flattening, and cognitive impairment [1]. The mainstay of treatment for schizophrenia involves the use of antipsychotic medications, which primarily function as dopamine D2 receptor antagonists, reducing excessive dopamine activity in the brain’s mesolimbic pathway to alleviate positive symptoms [2]. First-generation antipsychotics (FGAs), such as haloperidol, are highly effective in this regard but are often associated with significant side effects, including extrapyramidal symptoms (EPS) and tardive dyskinesia [3].

Second-generation antipsychotics (SGAs) are preferred in many cases due to their broader receptor profile, which typically includes antagonism of serotonin 5-hydroxytryptamine receptor 2A (5-HT2A) receptors in addition to dopamine D2 receptors. This broader spectrum of activity allows SGAs to target both positive and negative symptoms while minimizing the risk of EPS [4]. Among SGAs, aripiprazole has a unique pharmacological profile: it acts as a partial agonist at dopamine D2 receptors, a characteristic that sets it apart from traditional dopamine antagonists [5]. This partial agonism means that aripiprazole can act as both an agonist and an antagonist, depending on the endogenous dopamine levels in different parts of the brain.

This dual mechanism makes aripiprazole useful for addressing both positive and negative symptoms in schizophrenia, but it can also introduce therapeutic challenges. In some clinical contexts, aripiprazole’s partial agonism may lead to insufficient dopamine blockade, particularly in areas with high endogenous dopamine activity, such as the mesolimbic pathway, potentially resulting in a worsening of psychotic symptoms [6]. When combined with a traditional dopamine antagonist, such as haloperidol, the interaction between these medications can complicate treatment outcomes, as seen in this case report.

In this case, we discuss the complex pharmacodynamic interaction between aripiprazole and haloperidol in a patient with chronic schizophrenia who experienced a significant exacerbation of psychotic symptoms despite escalating doses of both medications. This highlights the importance of understanding the unique properties of antipsychotics when managing complex cases of schizophrenia.

## Case presentation

We present the case of a 44-year-old single Middle Eastern male who was brought to the emergency department of a tertiary psychiatric facility by a family driver due to escalating psychotic symptoms. He had been exhibiting hallucinatory behavior and expressing homicidal intentions for the past three weeks, during which time he had been wandering around the house with a knife. This occurred against the backdrop of a decade-long history of chronic schizophrenia, characterized by numerous admissions to our psychiatric facility and consistent non-compliance with his prescribed medications.

At the time of admission, the patient’s home medications included olanzapine 10 mg once daily (OD) (at bedtime {HS}) and aripiprazole 10 mg twice daily (BID). Upon further questioning, the patient revealed experiencing command auditory hallucinations, urging him to commit acts of homicide. He also disclosed persecutory delusions, primarily directed toward certain family members, particularly one of his brothers.

Given the severity of the symptoms and the risk posed to both the patient and others, he was admitted to the psychiatric ward. Upon admission, adjustments were made to his medications. Olanzapine 10 mg HS was discontinued due to concerns about the patient’s uncontrolled diabetes. The dosage of aripiprazole was increased to 20 mg once daily (OD), with the plan to transition him to a long-acting injectable (LAI) formulation to address his persistent non-compliance with medication and limited insight into his condition.

Over the next two weeks of inpatient observation, the patient’s hallucinations persisted, and he began to express homicidal ideation toward fellow patients. Despite increasing aripiprazole to 30 mg OD, there was no improvement in his symptoms. Haloperidol 10 mg HS was subsequently introduced to control his aggressive behaviors.

Unfortunately, the patient’s condition further deteriorated. He began to act upon his hallucinations and homicidal thoughts, displaying physical aggression toward fellow patients. On one occasion, while in the common room watching television, he suddenly stood up and punched the TV screen, causing significant damage. When questioned, he explained that he believed his thoughts were being broadcasted on the television channel. During this period, the patient also started expressing suicidal ideation, stating he intended to hang himself using his clothing or stab himself with the knives provided during meals. Haloperidol was subsequently increased to 15 mg BID in an attempt to better control his symptoms.

Despite these medication adjustments, the patient’s aggression continued to escalate. He became increasingly agitated, making multiple attempts to attack staff and security personnel. During one such outburst, the patient punched his hand and leg through a wall and door, resulting in a boxer’s fracture in his right hand, confirmed by an X-ray.

After 14 days of oral aripiprazole 30 mg OD, the decision was made to initiate the long-acting injectable (LAI) aripiprazole at 400 mg intramuscularly (IM). However, contrary to expectations, the patient’s aggression worsened within the next few days. He continued to engage in violent outbursts, and a particularly severe incident occurred when he punched a security guard in the eye, causing a serious injury.

In light of this alarming escalation, the treatment team conducted a thorough review of his medications. Both the LAI and oral forms of aripiprazole were discontinued. The patient’s regimen was adjusted; procyclidine, risperidone, valproic acid, and quetiapine were introduced and gradually titrated to their respective doses of 5 mg BID, 4 mg BID, 500 mg BID, and 50 mg HS, respectively. Haloperidol 15 mg BID was continued.

Throughout this period, it was important to consider the potential cardiac side effects associated with his antipsychotic regimen. Given the known risks of QT prolongation and arrhythmias with medications such as haloperidol, risperidone, and quetiapine, the patient was closely monitored with ECGs after regular intervals for any signs of cardiac abnormalities. Despite these considerations, the patient exhibited no cardiac issues during his hospital stay.

Over the following two weeks, the patient’s condition gradually improved. He became less physically and verbally aggressive and was observed spending most of his time watching television and interacting calmly with selected fellow patients. The team then began to taper the haloperidol dose to 10 mg BID and increased quetiapine to 200 mg HS.

Upon discharge, the patient’s medication regimen included procyclidine 5 mg BID, risperidone 4 mg BID, valproic acid 500 mg BID, haloperidol 10 mg BID, and quetiapine 200 mg HS. A follow-up appointment was scheduled for one week after discharge, followed by another at the one-month mark.

During both follow-up visits, the patient showed positive progress, with no concerns reported by his family. However, despite his improvement in terms of aggression and psychotic symptoms, he continued to struggle with maintaining personal hygiene and had limited social interactions, still requiring assistance with daily tasks. There were no further reports of violent behavior, and the patient appeared to be functioning at his baseline level.

## Discussion

Schizophrenia is primarily driven by disruptions in neurotransmission, particularly involving the dopaminergic system. The hyperactivity of dopamine in the mesolimbic pathway is associated with positive symptoms of schizophrenia, such as hallucinations and delusions, while deficits in the mesocortical pathway contribute to the negative symptoms [1]. Antipsychotic medications target these pathways by modulating dopamine receptor activity. Haloperidol, a first-generation antipsychotic (FGA), is a potent dopamine D2 receptor antagonist, effectively controlling positive symptoms but often causing significant side effects such as extrapyramidal symptoms (EPS) due to its strong dopamine blockade [2].

In contrast, aripiprazole, a second-generation antipsychotic (SGA), functions as a partial agonist at dopamine D2 receptors. This unique mechanism allows it to act as both an agonist in areas of low dopamine activity and an antagonist in areas of excessive dopamine, such as the mesolimbic pathway [4]. However, this dual action introduces complexity in therapeutic management, particularly when used in combination with antipsychotics that act solely as antagonists, such as haloperidol.

In this case, the patient’s psychotic symptoms worsened after combining haloperidol with aripiprazole. Aripiprazole’s higher binding affinity for D2 receptors likely displaced haloperidol, which functions as a pure antagonist. Consequently, aripiprazole’s partial agonist effect may have resulted in inadequate dopamine blockade, contributing to the patient’s worsening aggression and psychotic symptoms [5]. This phenomenon is well documented in the literature, where aripiprazole’s partial agonism has been shown to paradoxically exacerbate positive symptoms under certain conditions [6].

Furthermore, pharmacokinetic interactions between aripiprazole and haloperidol should be considered. Both drugs are metabolized by cytochrome P450 enzymes, particularly CYP3A4 and CYP2D6. When co-administered, these drugs can compete for metabolic pathways, altering their plasma concentrations and potentially leading to unpredictable effects on efficacy and safety [3]. Although plasma drug levels were not measured in this case, the interplay between these medications may have contributed to the patient’s clinical deterioration.

This case aligns with existing literature documenting adverse interactions between aripiprazole and haloperidol. A previously reported case involving a 39-year-old patient demonstrated a significant exacerbation of psychosis when aripiprazole was combined with haloperidol following prior risperidone treatment. It was suggested that aripiprazole’s partial agonist activity, when used alongside a pure antagonist such as haloperidol, can result in unpredictable clinical outcomes, similar to what was observed in this case [6].

Another important aspect to consider is the potential upregulation of D2 receptors caused by chronic haloperidol use. Long-term exposure to haloperidol can increase the sensitivity of dopamine receptors, enhancing the dopaminergic effects when a partial agonist such as aripiprazole is introduced. This receptor upregulation may have contributed to the worsening of the patient’s symptoms [7].

Finally, clinicians should also be aware of the potential cardiac risks associated with combining these medications. Reports have shown that the co-administration of aripiprazole and haloperidol can lead to a significant corrected QT (QTc) prolongation, highlighting the importance of regular ECG monitoring when these drugs are used together [8]. Although this particular patient did not experience cardiac issues, the possibility of such side effects should be considered, especially when managing multiple antipsychotics.

This case underscores the complexities of treating schizophrenia with multiple antipsychotics, particularly when combining drugs with conflicting pharmacodynamic properties. In this case, discontinuing aripiprazole and continuing treatment with haloperidol, risperidone, and other antipsychotics resulted in clinical improvement, suggesting that antipsychotics with more straightforward dopamine antagonism may be more appropriate in certain clinical contexts [5].

## Conclusions

This case highlights the potential challenges of combining antipsychotic medications with differing mechanisms of action, such as aripiprazole and haloperidol. Aripiprazole’s partial agonism at dopamine D2 receptors can complicate treatment, particularly when used alongside potent dopamine antagonists. Clinicians should remain vigilant for signs of worsening psychosis and carefully monitor patients receiving such combinations. The regular monitoring of plasma drug levels and cardiac function, such as ECG for QTc prolongation, is advisable, especially for patients on multiple antipsychotics.
